# From institutionalization of user fees to their abolition in West Africa: a story of pilot projects and public policies

**DOI:** 10.1186/1472-6963-15-S3-S6

**Published:** 2015-11-06

**Authors:** Valéry Ridde

**Affiliations:** 1 Department of Social and Preventive Medicine, University of Montreal School of Public Health, Montreal, Québec, Canada; 2 University of Montreal Public Health Research Institute (IRSPUM), Montreal, Québec, Canada

## Abstract

This article analyzes the historical background of the institutionalization of user fees and their subsequent abolition in West Africa. Based on a narrative review, we present the context that frames the different articles in this supplement. We first show that a general consensus has emerged internationally against user fees, which were imposed widely in Africa in the 1980s and 1990s; at that time, the institutionalization of user fees was supported by evidence from pilot projects funded by international aid agencies. Since then there have been other pilot projects studying the abolition of user fees in the 2000s, but these have not yet had any real influence on public policies, which are often still chaotic. This perplexing situation might be explained more by ideologies and political will than by insufficient financial capacity of states.

## Introduction

In this article, we present the general context of healthcare access in West Africa and interventions that have been aimed at improving access. Over and above the issues of geographic barriers and quality of care that are part of the long list of determinants of healthcare use [1], one of the greatest obstacles to access to care remains people's capacity to pay. Clearly, this article is not meant to minimize the importance of non-financial barriers [2], but in the context of this Supplement, which is focused on recent user fee abolition policies, our presentation of the general background is oriented toward issues of financial access. In the history of attempts to remove this financial obstacle, the train that has been rolling for the past 30 years has always been made up of two types of cars: pilot projects and public policies. These two processes of very different scopes-the first at the local level and most often organized by international aid agencies or researchers, the second at the national level and organized by the State-are sometimes sequential and, at other times, overlapping and interconnected. In this article, we consider that the formulation of public policy results partly from a sustainability process, notably through actions implemented as pilot projects, for which a key outcome is the institutionalization of policy instruments [3,4]-in this case, related to user fees. Pilot projects are understood here as being innovative interventions whose aim is to produce evidence and to influence scale-ups through the formulation of public policy [5].

Our aim in this analysis is to show that this interconnection has not yet been sufficiently effective, such that current user fee abolition policies are still not able to improve equity of access to the healthcare system. This article does not present a systematic review of the literature on this topic, as several such reviews have already been published in recent years [6-8]. Rather, our more modest aim is to provide an overview of the general background from a diachronic perspective, based on a narrative review [9] of the scientific and grey literatures.

## The disappearance of consensus around user fees

Let us begin with an image, that of the zombie. If this image is more familiar to readers of Haitian history than to those interested in public policy in West Africa, it is because the writings of certain health economists have not yet penetrated all borders. Robert Evans, a well-known Canadian economist, has used the zombie image for the past 20 years in talking about user fees. Despite the fact that the scientific evidence that should 'kill' this method of payment is very solid, it does not seem to want to die, surfacing time and again in political discourse and proposals, like the living dead [10]. Against all evidence [7,11], some health economists working in West Africa continue to assert that user fees have no influence on households' capacity to go to a health centre, or that recent fee exemption policies do not benefit the poor [12,13].

However, this appears to be a minority opinion, as an analysis of 120 documents published by 50 actors in the global health sector between 2005 and 2011 shows that almost no one now supports user fees [13]. The 1980s trend (not a consensus) toward user fees, due largely to strong international influence [14] and decision-makers' pragmatic need to find a solution to dramatic reductions in public funding for health [15], has thus been completely reversed. Even the economist who authored the notorious 1985 World Bank report that triggered waves of generalized user fees in Africa in the 1980s and 1990s has since changed his position [16]. In 2012, he called for the principle of user fees to be overturned [17]. Likewise, the president of the World Bank asserted, at the World Health Assembly in May 2013, that it is time to abolish user fees, whose existence, in his words, is "unjust and unnecessary". Some might think these changing trends are simply the latest fad. However, user fees are a technical instrument about which there is clear evidence. If there is any fad involved, it has more to do with organizations' readiness to accept this evidence than with the current state of knowledge about user fees.

Along the same lines, 15 years ago some authors hypothesized that public policies targeting the population at large, with no specific measures for the worst-off, would primarily benefit the most affluent [18]. This is, in fact, a significant issue, as the most recent fee exemption policies target broad social groups (pregnant women, children under five, etc.) universally. Recently that author, too, refuted his own hypothesis. He showed that, on the contrary, it is most often in countries where the coverage of interventions (such as assisted deliveries) has expanded most rapidly that inequities of access have been most reduced [19]. In other words, effectiveness was not achieved by sacrificing equity. Just as the thinking of certain economists has evolved, so too has that of West African policy-makers. When we initiated the research program (2009) presented in this Supplement, the great majority of those in authority were still in favour of user fees and very reluctant to abolish them. Today, however, it is very rare to hear anyone proclaim loudly and clearly that they are against eliminating user fees. Of course, this does not mean that behind closed doors, or in small committees, the resistance to eliminating user fees has evaporated. Publicly, however, no one appears ready to call for re-institutionalizing user fees.

This transition from user fees to their abolition is due in part to the failure of public policies in the 1980s and 1990s to improve healthcare access for the greatest number of people.

## Ineffective public policies around user fees and exemptions

The start and end points here must be service utilization and financial protection of the population, as these public policies target primarily those proximal objectives, which are also the objectives of universal health coverage [20]. Here we should look again at the situation as it was before the institutionalization of user fees in West Africa, as that situation is often forgotten or idealized. Decision-makers in health ministries and their partners in these regions generally consider the utilization rate of curative consultations in healthcare to be one of the most significant indicators of health system access. This rate, which is the ratio between the annual number of consultations and the number of inhabitants in the country, thus illustrates, to some extent, the level of accessibility from the health centres' standpoint. The World Health Organization (WHO) considers that, for the population as a whole, there should be five curative consultations per year per inhabitant [21]. We also know that a child will have, on average, around four health problems a year that require the use of modern health services [22]. In the 1980s, these utilization rates ranged from 0.09 to 0.23 per year in Benin, Guinea, Niger, Burkina Faso, and Mali [23]. In other words, in Benin a person would use a health centre once every 11 years, and in Burkina Faso, once every four years. Clearly, these utilization rates should be used with some caution, as the denominators are often poorly defined [24] and, as such, these rates only measure a fraction of real utilization of health services available. Some of the key explanations put forward for these low service utilization rates were the dysfunctionality of the healthcare system, its lack of funding, the continued levying of informal payments, and the lack of drugs in the health centres. "*For patients, the alternatives were simple. Whether or not services were officially free, if they had no money, they were not looked after, and if they did have some, they occasionally were*" (authors' translation) [25, p. 185]. As such, WHO and UNICEF encouraged the African States to adopt a so-called cost-recovery policy, known as the Bamako Initiative (BI). Even the Marxist-Leninist Mozambique of the late 1970s undertook a reform to introduce user fees [25]. UNICEF's idea was to replenish health centres' supplies of essential generic drugs (EGDs) so that patients could be treated at lower cost. However, to ensure the sustainability of the EGD supply, it was decided that patients would be asked to pay user fees directly to the health centres, which would retain this income locally (contrary to what sometimes occurred before then, which was that payments would be centralized and absorbed into the administrative maze, as they represented only 5% of the overall health budget [26]). This income, managed by a committee of local representatives to reinforce the community-based approach and local 'governance', was to be used to replenish EGD stocks. The profits were intended to cover certain minimal expenses associated with the centre's activities, but also to improve service quality and equity. In addition to selling EGDs, some countries decided, at the outset or later on, to charge fees for medical acts as well. As such, in the 1990s, all West African countries were involved in implementing this partial cost-recovery policy.

Five years after this policy was implemented, studies led and funded by UNICEF noted that service utilization rates had risen. They were multiplied by factors of 1.5 to 5.6, depending on the country, or between 0.16 and 0.31 new consultations per year per person [23]. Yet in the best case at that time (Benin, with 0.31), this represented just one consultation every three years. In Niger, the institutionalization of user fees in 1995 reduced the number of new cases treated in the district of Tillabery by 40% [27]. That study confirmed the results of the 1993 pilot project in Niger, in which a 32% drop in consultations was observed among the poor after user fees of 200 F CFA (USD 0.27) were introduced [28]. In Burkina Faso, the introduction of user fees for consultations in 1997, on top of the fees for drugs, reduced utilization by 15% in the district of Kongoussi [29]. In 1993 in Niger, only 11% of *poor and sick *persons went to a health centre; in Burkina Faso that proportion was 17% in 1994, and in Senegal, 29% in 1991 [30]. By 2010, 20 years after that reform, there had been almost no change in these rates, which ranged from 0.30 in Guinea to 0.36 in Burkina Faso. In other words, health services were not being used, or just barely, making those health systems inefficient [31]. Moreover, a multi-country study showed that, in the context of user fees, health centres were used more by the well-off than by the poor [32]. On top of that, when exemptions were implemented in this cost-recovery system, more than 70% of those were monopolized by the non-poor [30]. Today, most national surveys in the West African region confirm this low utilization. Less than half of *sick *children go to a health centre for care (see all the Demographic Health Surveys - DHSs). Even in Rwanda, often cited as an example of success in Africa, only one-third of sick children go to health centres, in a context where health mutuals (which cover 85% of the population) demand a co-payment at the point of service [33].

One of the key achievements of these policies consisted of bringing drugs back into health centres, especially in countries that were able to organize a drug supply system and an effective national purchasing agency. However, this was not the case everywhere; in some countries, such as Niger and Ivory Coast, the central purchasing agencies were very ineffective [34]. This presence of drugs in health centres was the only successful outcome of the BI, which failed in all other respects. Community participation and the populations' reinforced role in health centre management most often did not work out well, as observed in Senegal, Mali, Niger, and elsewhere in Africa [35,36]. Health centre personnel made most decisions and had increasing power over community-based management committees. So-called community participation most often referred only to users' financial participation, a situation deplored by those at UNICEF who originally conceived this policy [37]. The fees collected from patients who were able to pay for treatment at health centres were hoarded away and never used to improve equity of access to care. The profits on drug sales were sometimes well above official norms, as was health workers' share of user fees [38]. Lastly, the most flagrant failure of these policies concerned the care of indigents, or exemption from user fees for the worst-off, according to the vocabulary of that time and Principle 7 of the BI. While this measure was intended from the outset-even if today UNICEF promoters insist that was not the case [39]-no country has wanted to grapple with this issue, and indigents have remained at the margins of healthcare systems [40].

All of these failures to improve service utilization, taken together, led stakeholders involved with user fee policies to modify their views. They reached the point, after South Africa in 1994, of wondering whether one solution might be to simply abolish user fees altogether. Thus, key leaders of African States, the European Commission, the United Nations, and the African Union called for abolition [41,42]. Other articles in this supplement describe these policies, so we will not go into the details here. However, for the historical reflection proposed here, which requires a broader perspective, we should remember that the impacts of exemption policies in Africa have, overall, been modest. Today they appear to be less substantial than decision-makers were expecting.

Of course there are methodological issues, since it is difficult to attribute changes to policies in contexts where decision-makers do not take methodological issues into account in their decisions, as a recent World Bank international study on universal coverage also concluded [43]. Reviews of the literature on these impacts reveal all the shortcomings of the research methodologies used, which were most often based on data from health centres, employed statistical methods that were not sophisticated enough for causal attribution, examined time frames that were too brief, or did not have sufficiently robust control groups [44,45]. These weaknesses are, of course, inherent in all evaluation processes examining public policies in natural contexts. However, these studies also highlight the desperate lack of rigorous data on the impacts of these policies in Africa, even if the trends regarding their positive impacts all point in the same direction [44,45]. In Burkina Faso, for instance, statistical modelling demonstrated that the lives of 14,000 to 19,000 children under five could be saved if a fee exemption policy that was shown to be effective in one region were to be scaled up nationally [46].

Let us consider the example of one country where the needs are among the greatest in Africa: Niger, for which three robust studies have provided very useful data. A first study showed that interventions targeting child survival (free care policy, bed net distribution, malnutrition reduction program) saved nearly 60,000 lives compared with the situation in 1998 [47]. However, with their methodology it was not possible to isolate the impacts of the free care policy because several major interventions were implemented concurrently. A second study carried out in four districts in Niger concluded that, in 2009, 70% of *sick *children went to a health centre, whereas that proportion had been only 14% before the fee exemption [48]. A third study described the rise in health centre utilization after the implementation of free care for children. Although the rise was immediate and substantial (+98%) when the policy was implemented country-wide, it remained at that level until the end of the study period in December 2008. At the same time, however, there was considerable heterogeneity in the changes in the different districts; for example, four of the 11 districts studied were not able to sustain the immediate impacts on utilization after 18 months of implementation, but utilization stagnated in only one of those four [49]. These three studies thus indicated that the situation for Nigerien children had improved greatly during the first two years of the policy's implementation. However, given the major problems that country encountered in implementing the exemption policy after 2009 (Diarra & Ousséini, this issue), this success must be reconsidered in light of the fact that most of the health centres subsequently declared bankruptcy [50]. Sierra Leone, where nearly 85% of sick children were able to go to a health centre after three months of fee exemptions, compared to 45% previously [51], appeared to be experiencing the same situation. In Zambia, the initial rise in service utilization (+32%) eroded over time, settling at only 19% after 18 months of implementation [49]. Impacts were definite but short-term, as the policy ran out of steam, problems arose in implementation, and drug shortages intensified, particularly due to lack of funds to support the policy [52], or to such funds being purely and simply diverted, as was the case in Sierra Leone [53]. In Zambia, the exemption policy did not seem to have affected the availability of drugs [54], and, in any case, drug shortages occurred regardless of whether or not the health centres applied the exemption [55]. In Mali, the policy providing free malaria treatment to children led to only a 30% increase in consultations [56], and the rise in the number of caesareans after medical acts and kits were made free was also modest but significant in one region (Kayes) for women living in cities with hospitals [57]. However, that study also showed that the predicted probability of survival for mothers and newborns was improved (on the order of 10 percentage points) after free caesareans were introduced, in both cities and villages. These results take into account factors that might influence that probability, such as the women's ages, the district, clinical indications, history of caesareans, and existence of a referral-evacuation system. However, women continue to pay a heavy financial price for caesareans, and between 20% and 50% of households still face catastrophic expenses [58,59]. These few examples testify to the currently limited impacts of exemption policies in Africa. It would be just as easy to show numerous other cases where the impacts are not limited, but simply non-existent or destabilizing, as is the case for the elderly, children, and parturients in Senegal [60,61], adults in Zambia [49], or even pregnant women and women in Sudan or Ethiopia [62,63].

On the other hand, two counter-examples can be presented to show that West African States are, in fact, able to find the means to make their exemption policies work. Both have to do with childbirth policies in two neighbouring countries.

First, in Ghana, the fee exemption policy for deliveries implemented in 2005 and its subsequent incorporation into the national health insurance contributed greatly to the increase in facility-based deliveries with qualified personnel. By the end of 2009, more than 70% of deliveries occurred in maternity units, according to information produced in seven health districts. This exemption policy also reduced inequalities in utilization, as it was women from the poorest households who benefited most. Of course, the policy alone could not eliminate these inequalities, but it helped to reduce them. The gap in assisted delivery rates between the least poor and the poorest went from 65% in 2004, before the first policy, to 54% in 2009 [64].

Next, we turn to the national policy to subsidize assisted deliveries and caesareans in Burkina Faso. This policy's impacts have been impressive and could serve as proof to refute the hypothesis that West African States are incapable of seriously organizing a public policy. In some of the country's rural districts, with no external partner involvement, nearly 90% of women today deliver in the maternity unit of a health centre with qualified personnel. Expenses related to deliveries have also been substantially reduced [65,66]. However, while these changes definitely qualify as successes in comparison with other countries in the region, numerous limitations should be noted. First, this is not a user fee abolition policy, but rather a subsidy, as the women still have to pay 20% of the costs as estimated by the Ministry of Health of Burkina Faso (i.e., USD 1.25). Then, total exemption from payment, funded by the State and adopted by the Parliament (without dedicated outside funding), was planned for indigent women. However, this exemption has never been applied, nor has any support ever been provided to health workers to ensure this measure's application (most are not even aware it exists), even though the funding is available [67]. Lastly, there is a persistent discrepancy in the implementation, since numerous studies have shown, in several districts of the country, that over half of the women paid more than the standard fee required [65,68,69]. Thus, in a context where the financial and human resources are all in place to support the policy's implementation, parturients continue to be victims of improper implementation, and inequalities between the poor and others persist, whereas they could have been mitigated if the funding voted for indigent women had been used as intended.

There is thus an implementation problem that explains, to some extent, the ineffectiveness of policies in West Africa (Olivier de Sardan, this issue). The user fee policies of the 1980s and 1990s made it possible finally to have drugs on hand locally, but only for people with the capacity to pay. Community participation, care for indigents, and quality of care (aside from drug availability) were neglected. The exemption policies of the 2000s have significantly improved access to care, but they are difficult to sustain, their implementation is chaotic, their funding is inadequate for the needs, and they have not yet managed to reduce the inequities of access to the healthcare system. And yet, whether for user fees or their abolition, a great deal of scientific and experiential knowledge was available to ensure the policies' design and implementation would be effective and equitable. Many pilot projects had been conducted in West Africa whose results could have been used to good effect, which was not done. Of course, as shown by Olivier de Sardan et al. (this issue), there are numerous challenges associated with scaling up and funding these policies and with using the knowledge from these pilot projects [5,70], to which we will return. We will show this in the next section before putting forward, in the conclusion, some explanations for this problematic knowledge transfer process at a time when there is a growing trend toward having evidence-based medicine represented at the public policy table [70].

## Pilot projects that are essential yet unused/unusable

When UNICEF launched the cost-recovery policy (WHO being opposed to this initiative at that time [37]), some questioned how it was possible to proceed so quickly when the new policies were based only on a few pilot experiments: "*It is dangerous to jump from two small projects to a multimillion dollar enterprise*" [71]. In Niger and Burkina Faso, experiments had been organized and driven by American aid agencies (USAID); in Benin, Guinea, and Mali (in particular) by UNICEF; and in Cameroon by German aid agencies [23,28,72]. Subsequently these experiments greatly influenced the political agenda toward selecting user fees as a solution for improving access to care. Lee and Goodman [14], in analyzing this period, state that "*in the area of HCF [health care financing], a global elite has come to dominate policy discussions through their control of financial resources and, perhaps more importantly, control of the terms of debate through their expert knowledge, support of research, and occupation of key nodes in the global policy network*." These pilot projects most often showed that the imposition of user fees, associated with the presence of drugs (which promoters took as an approximate measure of improvement in care quality), had made it possible for many more people to attend health centres. As we saw above, these utilization levels, while often higher than before, were nevertheless very low, and remained low 20 years after the fact. A synthesis article showed that some pilot projects even asserted that quality improvements could offset the negative impact of introducing user fees [73]. In Cameroon, for example, user fees without any quality improvement had negative impacts on the use of services by the worst-off. However, those authors showed that better availability of drugs in the health centres made up for the negative impacts of user fees [72], even for the worst-off, a result also seen in an experience in Niger [28]. On the other hand, other studies then explained that the links between price, quality, and utilization are more complex than that, and that the different aspects of each of these dimensions can affect outcomes. Quality improvement does not automatically compensate for negative effects of higher prices on service utilization [73].

Moreover, none of these experiments in the 1990s focused seriously on access to care for indigents, on attempts at cross-subsidization, or on exemptions [40,74]. Even in the early 2000s, when a non-governmental organization (NGO) project funded entirely by the European Union was set up for three years in northern Burkina Faso to help this health region organize cost recovery (whose shortcomings had been known for years), the issue of indigence was completely neglected. It was considered too complicated and not a priority by all the project's stakeholders, who were focused on geographic access to drugs [75]. Moreover, one of the few studies undertaken to understand why this issue was not addressed showed that there was no window of opportunity nor any political entrepreneur available to propose solutions for indigent access to healthcare, which, in any case, was not considered a public problem [76]. One of the few NGOs interested in the issue of indigence in Africa [77], funded by UNICEF at the time, abandoned this issue. It gave up its attempted interventions for the worst-off, deciding instead to invest in supporting community-based health insurance plans, which never bother with the indigent [78].

However, these experiments were all largely controlled by major international actors, and any success they had was due to the personal motivation of local managers [79,80]. The problems they experienced in responding to population needs, the financial barrier created by user fees, and the lack of consideration for equity were never actually taken into account when public cost-recovery policies were being established and implemented. It was simply decided that these should be organized, and it was done, regardless of any scientific evidence about their difficulties and deficiencies. A little later in the narrative, near the end of the 2000s, the few, very rare operational studies undertaken to incorporate indigent care into cost-recovery systems revealed shortcomings of their own [81,82]. In Burkina Faso, studies indicated that rural communities could be effectively mobilized to select indigents [83,84] without causing social stigmatization [85]. However, at the same time, those studies highlighted rural households' feeble capacity to contribute financially to providing care for the worst-off. In other words, considering the poverty of rural areas, it did not seem feasible to expect the poor, who rarely use paid services at health centres, to fund, by means of user fees, access to free care for the indigent. It was difficult to ask the poor to fund exemptions for the very poor. The principles of the BI, under which payments were required from some patients while others were exempted at the most decentralized levels of the healthcare system, thus came up against the reality of households' contributive capacity. The issue of the losses generated by these exemptions and whether they should be covered centrally by the State is fundamental, but has rarely been considered. This is why, all along, and most recently through WHO and the movement for universal coverage [20], there has been a push to fund healthcare based on the principle of prepaid contributions shared at the national level and adapted to people's capacity to pay.

But before these principles were given concrete expression, or any political will was felt, as happened in Ghana, other pilot projects were conducted in the mid-2000s in certain West African countries that had not engaged heavily in exemption policies. Most often these projects experimented with fee exemptions, not only for the indigent, but also for categories of people considered vulnerable (children, pregnant women, etc.), and most often directly in line with Millennium Development Goals (MDGs). This was the case in Burkina Faso, Mali, Niger (see 4th section below), as well as in Ivory Coast, Guinea, and the Democratic Republic of Congo [86-88]. As in the cost-recovery projects, these were aimed at demonstrating the principle, but in the opposite direction, i.e., showing the effectiveness and equity of user fee abolition. In all cases, the outcomes were conclusive, but the magnitude of the increase in utilization was beyond comparison with the experiments of the 1990s. Utilization reached record highs for such data, to our knowledge. In these pilot projects, utilization was so great that most often, after a few years, it neared optimal satisfaction of population health needs. Utilization rates were nearly three (sometimes more) consultations per year per child. More than 80% of *sick *children went to health centres, and almost all women gave birth in maternity units [89,90]. The weight of the financial barrier in determining utilization was clearly demonstrated as, most often, these experiments did not address geographic barriers to care. Like the cost-recovery projects, these experiments were largely under the control of international organizations (with a greater preponderance of NGOs, compared with the 1990s), but with a certain heterogeneity of processes. On one side were the NGOs 'without borders', whose actions followed a substitutive approach; this was the case in some districts of Mali, in Guinea Conakry, and in the Democratic Republic of Congo. On the other side, some NGOs attempted to integrate their projects into the healthcare system and essentially took on the role of third-party payers who paid health centres on behalf of patients, as was done in Burkina Faso.

However, what is most remarkable is that the lessons derived from these pilot projects of the 2000s were no more taken into account in public policies than the results of the 1990s cost-recovery projects had been. In Niger, for example, the State drafted its free care policy for children under five without taking into account the results of a pilot project, despite the fact that they were well known and had been studied by the authorities [91]. In Mali, a pilot project showed that making drugs free without removing user fees for consultations did not adequately satisfy population needs [92]. Nevertheless, the State followed this principle in drafting its policy (Touré, this issue), with the result that the outcomes were limited. Trials in Burkina Faso showed that many more women used maternity units when deliveries were free than when they were only subsidized [93], and a study showed that the budget voted by the National Assembly for this policy was enough to organize such an exemption [67]. Yet the State continued to require women to pay, to 'participate' financially, with the stated aim (debunked by scientific evidence [11]) of making people 'responsible' [94].

Still, Burkina Faso did occasionally take into account the results of pilot projects, as Ghana did when decision-makers decided to scale up the free delivery policy to the national level after a pilot in four regions [95]. For instance, in Burkina Faso, trials of cost-sharing for caesareans in some districts of the country [96] enabled the authorities to estimate the caesarean costs that needed to be taken into account in the emergency obstetrics and newborn care (EmONC) policy. They also decided, in a move that was quite unusual in the region, to cover the full cost of transportation between the health centre and the district hospital for emergency obstetric cases. Yet this remains an isolated example, as most often decision-makers in Burkina Faso have no desire to carry out pilot projects to inform their decisions. We see this currently with regard to the introduction of pay-for-performance, which has been widely promoted by the World Bank. In the fight against malaria, the situation is even more disturbing. In fact, the national policy for home-based malaria treatment by community health workers (PECADO) was deployed country-wide without waiting for the results of pilot projects that the State itself had organized in three districts. Thus, Burkina Faso remains a baffling case of inconsistent choices by decision-makers, who most often say they do not want pilot projects (the case of pay-for-performance), but sometimes commission them (malaria), sometimes accept their existence (free care programs), but do not often take them into account (caesareans).

## The challenges of using pilot projects in drafting public policies

In theory, those drafting a public policy should take into account lessons derived from prior studies to ensure the policy is contextually appropriate and to avoid any deviation in its implementation, so that the objectives might be attained. However, the example of the institutionalization and subsequent abolition of user fees in West Africa seems to demonstrate that this is not done.

Scaling up any healthcare innovation, such as user fees or their abolition, is clearly not easy, as there are numerous factors to take into account, above and beyond the innovation's intrinsic instrumental components. Factors that require particular attention include the functioning of the healthcare system and its particular shortcomings, the characteristics of the organization promoting the pilot project, such as NGOs (perceived by the State as being too often focused on rights, social context and environment, on values related to equity, and on problems related to healthcare funding), as well as the scaling-up approach used during the course of the experiment [97].

How this scaling-up of fee exemption pilot projects is to be funded is certainly a core issue. However, more than being a question of means, this issue appears to us to be a matter of political will and priorities in the public agenda. The first thing to note is that almost no African countries give sufficient priority to the health sector. The objective of dedicating 15% of the State's budget to this sector (Abuja Declaration, 2001) is rarely met (13.6% in Burkina Faso, 10% in Mali, 11.1% in Niger) [98]. Yet national and international resources are often available. Certainly, we could mention here the new mining and oil revenues available to the three countries covered by our research program, even though access to information on this subject is limited. We could also, however, point to the example of Mali, where 31 billion franc CFA was allocated for Ministry of Health activities in 2009 from external (and therefore available) funding, but was never paid out [99]. As well, certain national resources normally allocated for the poor are sometimes misused. The 120 billion franc CFA released by the Burkinabè government during the 2008 crisis to help the worst-off actually benefited the more affluent [100]. This 120 billion franc CFA should be considered in comparison with the 2 billion franc CFA annual budget of the national childbirth subsidy program. The issue here is one of priorities and proper use of resources. In recent years, Ghana has had the political intent to increase its value-added tax (VAT) (which remains progressive [101]) to fund two-thirds of its national health insurance [95]. Meanwhile, in 2012, Niger and Gabon managed to mobilize 2 billion F CFA to send their football teams to the Africa Cup of Nations! Finally, more resources would be available at the international level if donor countries would respect their commitment to devote 0.7% of their gross national product to public development aid [102].

Thus, it makes little sense for certain NGOs or international aid agencies to set up pilot projects in Africa without bothering to adapt them adequately to States' capacities, beyond demonstrating a principle that might, in some situations, still be important to carry out. A better solution would be to adopt a stepwise approach in which pilot projects would be organized by and for the State. To some extent, this is what has been done in Burkina Faso over the past few years with regard to fee exemptions for children under five. Some NGOs and their funding agencies worked together to implement pilot projects that were increasingly integrated into the health system context (even to the extent, after five years, of asking the districts to manage the reimbursement of fees with the NGO's budget), while producing evidence that could inform national decision-making [103]. The State took the time to understand these projects (2008-2012), to carry out its own feasibility study for a national policy (2012), and to produce a well-argued strategic document (2013) that was submitted to the Ministry of Health. History (and research) will tell whether this process was helpful to the Burkinabè authorities in their decision-making.

However, we have seen that, for political reasons, decision-makers balk at the pilot project approach, in the name of equal treatment for all, but also because of the difficulty of explaining to citizens the value of such experiments. Nevertheless, even when these experiments are taken into account, if the evidence shows that user fees do not make patients more 'responsible', or that exempting people from paying for drugs but not for consultations has only limited benefits, decision-makers often have trouble considering these facts. Of course, we can challenge those naïve researchers who believe public policies should be based only on evidence, setting aside political concerns. On the other hand, we might also ask whether decision-makers' own knowledge and ideologies might not be impediments (that should be taken into account rather than ignored) to the formulation of appropriate public policies [104,105]. In the case of user fees, their ideas about the role of the market or of the State certainly underlie their understanding of the policy instrument. The economic framework has taken centre place in public policy formulation [106]. However, to those who read most of the documentation on national health policies and other national health development programs in that region, this comes as no surprise, as these documents are not based on any scientific references, whether local or international.

A recent study in another region of Africa (Uganda) than the one covered in this article appeared to show, conversely, that decision-making on user fees abolition policy was influenced by evidence [107]. However, it also showed that community complaints appeared to exert the greatest influence on decision-makers, yet these types of data are based on public opinion and not on information verified by research.

## Conclusion

There is thus a real discrepancy between the current state of knowledge on healthcare user fees and their abolition, on one hand, and how that knowledge is taken into account in public policies, on the other. Involving decision-makers as early as possible in reflecting on research priorities, implementing them and then applying results is certainly one approach that should continue to be promoted, even though it presents significant challenges [70]. It is tempting to think that the ways in which lessons from pilot projects are used do not follow any clear logic. It may also be, however, that this situation is caused by cognitive dissonance, in which only those lessons that reinforce the authorities' existing ideas are retained, while those that contradict their beliefs are dismissed. Thomas Kida [108] reminds us, indeed, that "*by selectively focusing on supporting information, we ignore contradictory information that may be very relevant to the decisions we make*." Pierre Muller quite rightly asked "*where does this idea come from, that a public policy expresses a sort of 'truth' of the moment, not based on the outcome of a scientific experiment, but because it corresponds to the actors' beliefs?" *(authors' translation) [109]. This opens up a new field of research, to our knowledge as yet unexplored in West Africa, which would study the links between researchers and decision-makers, between science and policy, and between ideas and evidence.

## Competing interests

The author worked as a consultant for NGOs that implemented user fee exemption interventions in Africa. The funders and NGOs did not take part in decisions on the study design, data collection or analysis, nor in the preparation or publication of this manuscript.

## Authors' contributions

VR conceived the idea, wrote the draft and final version of the manuscript.

## Declarations

This article has been modified from the chapter **De la généralisation à la suppression du paiement direct: une histoire de politiques publiques et de projets pilotes** by V. Ridde in the book *Une politique publique de santé et ses contradictions. La gratuité des soins au Burkina Faso, au Mali et au Niger*, J.-P. Olivier de Sardan and V. Ridde (eds), 2014, Karthala, Paris [110], with the permission of the publisher. The publication of this supplement was funded by a grant from International Development Research Centre (IDRC), Ottawa, Canada.

This article has been published as part of *BMC Health Services *Research Volume 15 Supplement 3, 2015: User Fee Exemption Policies. The full contents of the supplement are available online at http://www.biomedcentral.com/bmchealthservres/supplements/15/S3.
